# Point-of-care ultrasound findings in unselected patients in an emergency department *—results from a prospective observational trial*

**DOI:** 10.1186/s12873-018-0211-4

**Published:** 2018-12-27

**Authors:** Jesper Weile, Christian B. Laursen, Christian A. Frederiksen, Ole Graumann, Erik Sloth, Hans Kirkegaard

**Affiliations:** 1 Emergency Department, Regional Hospital Herning, Herning, Denmark; 2 Research Center for Emergency Medicine, Aarhus University Hospital, Palle Juul-Jensens Blvd. 161, 8200 Aarhus, Denmark; 3 Department of Respiratory Medicine, Odense University Hospital, Odense, Denmark; 4 Institute of Clinical Research, University of Southern Denmark, Odense, Denmark; 5 Department of Cardiology, Aarhus University Hospital, Aarhus, Denmark; 6 Department of Radiology, Odense University Hospital, Odense, Denmark; 7 University of Cape Town, Cape Town, South Africa

**Keywords:** Point-of-care ultrasound, Prevalence, Emergency medicine

## Abstract

**Background:**

Point-of-care ultrasound (POCUS) can improve patient management in the emergency department (ED). However, previous studies have focused only on selected groups of patients, such as trauma, shock, dyspnea, or critically ill patients, or patients with an already known diagnosis. Most patients seen in the ED do not match these criteria. We aim to present total prevalence of positive findings when basic POCUS is applied to the broad population of patients seen in an emergency department.

**Methods:**

We conducted a single-center prospective explorative observational study of 405 unselected patients aged 18 years or over. A structured whole-body ultrasound examination was performed on all patients within 2 h of arrival to the ED. The ultrasound examination consisted of focused cardiac ultrasound, focused abdominal ultrasound, focused assessment with sonography for trauma (FAST), and focused lung ultrasound.

**Results:**

We managed to perform 94.5% of all planned examinations. The study revealed positive findings in 39.3% of all included patients. This study presents the prevalence of positive findings among subgroups of patients. Divided among the categories of chief complaint, we found 62 positive examinations in 58 (14.3%; 95% CI, 10.9–17.7) unique patients with orthopedic complaints, 77 positive examinations among 59 (14.6%; 95% CI, 11.1–18.0) unique patients with medical complaints, and 55 positive examinations among 42 (10.4%; 95% CI, 7.4–13.3) unique patients with abdominal surgical complaints.

**Conclusion:**

POCUS revealed positive findings in more than one third of unselected patients in the emergency department. The study presents the findings and distribution among categories of chief complaints. Future investigations are necessary to elucidate the implication of the findings.

**Electronic supplementary material:**

The online version of this article (10.1186/s12873-018-0211-4) contains supplementary material, which is available to authorized users.

## Background

Timely and accurate diagnosis in the emergency department (ED) can shorten length of stay, decrease morbidity and mortality, prevent prolonged discomfort, and prevent adverse effects by initiating incorrect treatment [1]. Point-of-care ultrasound (POCUS) is a diverse, noninvasive diagnostic tool considered by many to be the modern form of the stethoscope [2, 3]. Ultrasound has been used in clinical medicine since the late 1950s [4]. with no proven clinically relevant harmful effects like with radiation from conventional X-ray or computer tomography (CT). POCUS can be performed by emergency physicians during initial work-up with adequate accuracy [5] and can increase overall diagnostic accuracy and alter patient management [6, 7].

Diagnostic workup in emergency medicine is heavily based on probabilistic reasoning [8]. Knowledge of the prevalence of any given finding is essential and affects the synthesis of diagnoses based on pre- and posttest probability. Previous ultrasound studies have examined selected groups of patients such as trauma, shock, dyspnea, or critically ill patients, or patients with an already known diagnosis. Furthermore, these studies have been designed for specific evaluations, such as of the heart or lungs [6, 9–14]. The usefulness of a whole-body POCUS examination on patients outside these selected groups, especially in the non-critically ill, is unclear.

The Danish heath care system is based on socialized health care. During the daytime, patients are referred by the GP or an out-of-hours GP, or are brought to the ED by ambulance. In case of an acute emergency, the patient can attend the ED without any referral [15].

The Danish health care system facilitates direct referrals for some specialties, thus bypassing the emergency department. These include patients suspected to have acute myocardial infarction, who are transferred directly to a catheterization laboratory, as well as stroke patients who are candidates for thrombolysis or mechanical thrombectomy, women in labor, and children with a presumed medical emergency.

No studies have investigated the prevalence of different POCUS findings in the spectrum of unselected patients presenting in the ED. The aim of this study is to report all POCUS findings in unselected patients in an ED and present the baseline prevalence of different positive findings.

## Methods

The study was a prospective, observational, single-center trial in Regional Hospital Herning, an urban 24-h general emergency department in Denmark. The department has an annual uptake of approximately 35,000 patients and serves a population of approximately 300,000 inhabitants.

## Inclusion of patients

Patients were included by convenience sampling when the primary investigator was present in the department. Inclusion criteria: All patients presenting in the ED who were older than 18 years of age, for whom an ultrasound examination could be performed within 2 h after arrival, with informed consent from the patient or patient’s next of kin and primary care practitioner.

We aimed to include patients in the same distribution as the background population in the ED. Including a sample of 400 was thought provide the capacity for subgroup analysis and was believed to be feasible within the available time frame. Furthermore, expanding the inclusion to 406 patients would enable a later substudy on changes in management. To our knowledge, no prior studies have provided sufficient knowledge for sample size estimation.

The inclusion process was designed to avoid selection bias and for the primary investigator to have no influence on which patients would be included. We performed a prestudy evaluation of the ED’s background population. We evaluated 815 visits during 11 days in May 2012 for the distribution of chief complaints. According to their initial chief complaint, the patients sorted into the following categories (for more information on the selection criteria for relevant categories, please see Additional file 1: Table S8):

- Orthopedic: 384 (47.1%)
- Medical: 242 (29.7%)
- Abdominal surgical: 189 (23.1%)

These groups correspond to the original sorting of patients before the EDs were established in Denmark after 2007. To enable the mirroring of this distribution, 406 nontransparent numbered envelopes were prepared. Each envelope contained a note reading “medical,” “abdominal surgical,” or “orthopedic,” with the percentages of each category corresponding to the findings of the pre-study evaluation. The sequence of the envelopes was randomized using the freely accessible random.org random sequence tool [16]. When initiating the inclusion of a patient, the primary investigator would open an envelope, mark the time of opening, and approach the next patient entering the department who would match the category revealed from the envelope.

## Ultrasound examinations

**Fig. 1:**
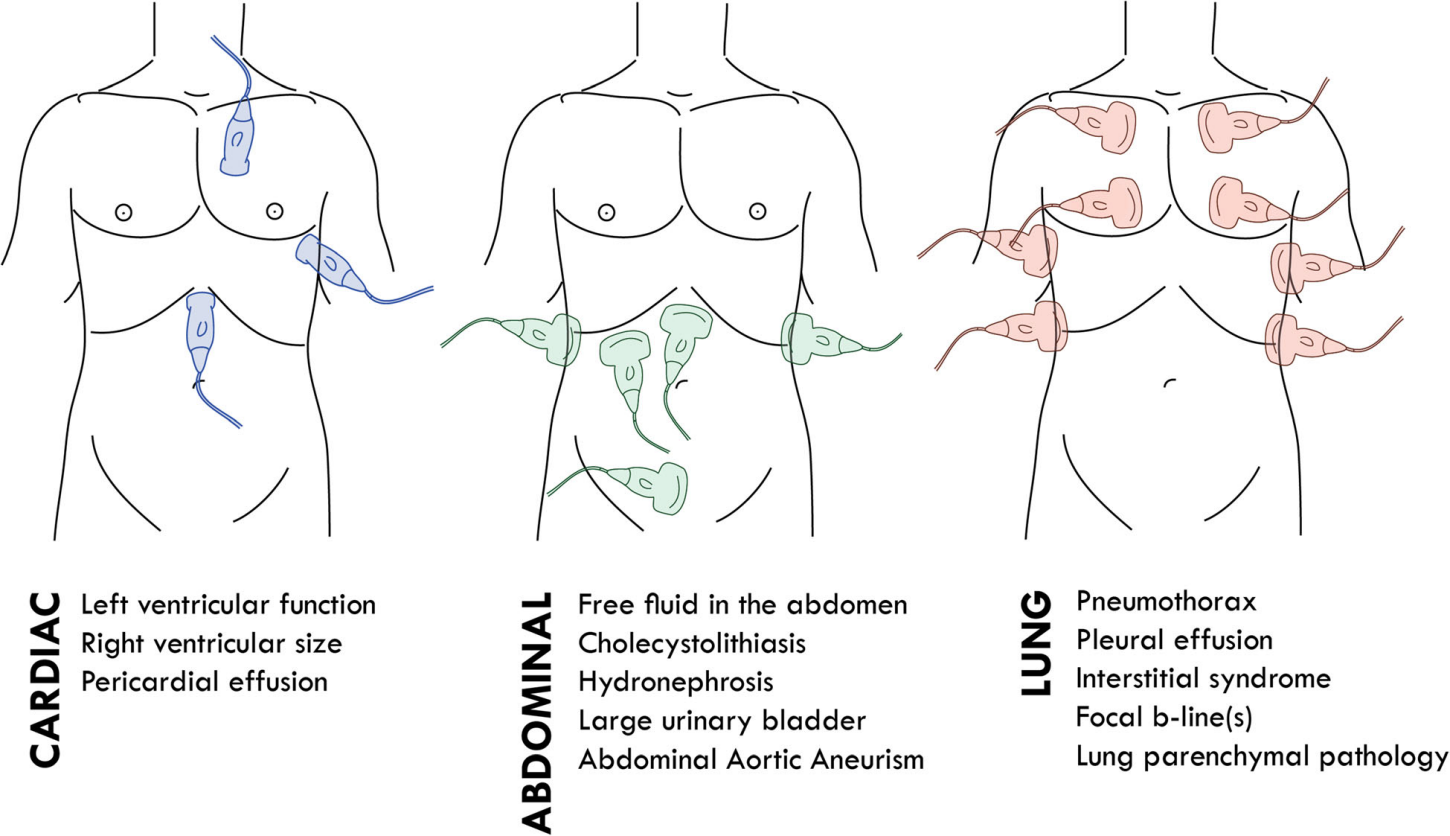
All Performed Ultrasound Windows and the Positive Findings Evaluated during the Study

The types of ultrasound examinations chosen for the study were based on the following considerations. First, all of the examinations are included in the International Federation of Emergency Medicine Point-of-Care Ultrasound Curriculum Guidelines [17]. Second, they comply with the “Framework for Implementation, Education, Research, and Clinical Use of Ultrasound in Emergency Departments by the Danish Society for Emergency Medicine,” published in 2014 [18]. Third, they are considered achievable to learn for physicians in the field of emergency medicine [19]. All windows reviewed and positive findings assessed are shown in Fig. 1.

Focused Cardiac Ultrasound (FoCUS): Four basic views where achieved: the subxiphoid four-chamber view, parasternal long-axis view, parasternal short-axis view, and apical four-chamber view. These views are the foundation of the most common focused cardiac ultrasound examination protocols [20–22], as well as the basic principles recommended in the emergency setting by the American Society of Echocardiography and the American College of Emergency Physicians [23]. The scans were assessed for 3 findings according to the International Federation of Emergency Medicine Point-of-Care Curriculum Guidelines [17]: pericardial fluid, dilated right ventricle, and left ventricular systolic contractility. The examination was considered positive for pericardial fluid if the pericardium had hypoechoic areas of above 10 mm. Right ventricle dilation was considered positive if the right ventricle/left ventricular end diastolic ratio was more than 0.6 [24]. Left ventricular systolic contractility was estimated with the eyeballing technique and categorized into the 5 following categories: hyper dynamic, normal, reduced, moderately reduced, or severely reduced [25].

Focus Assessment with Sonography in Trauma (FAST) Examination: We achieved the views defined at the International Consensus Conference in Baltimore in 1997 [26]: the right upper quadrant, left upper quadrant, and pelvic sagittal and transverse views. These windows were used for visualizing intra-abdominal free fluid. The subcostal view of the pericardium was also obtained; however, pericardial effusion was assessed during the cardiac examination. The FAST examination was interpreted as being positive for free fluid if anechoic areas of any size were observed. The FAST protocol was included because it is the most widespread ultrasound protocol used in emergency medicine [10].

Focused abdominal ultrasound (FAUS): The kidneys were visualized in a longitudinal plane, and a full sweep was recorded. The sweep was assessed for hydronephrosis or intrarenal nephrolithiasis using the eyeballing technique [27]. The scan was considered positive if large filling of the kidney pelvis was observed or if focused obstructions of sound causing hyperechoic surfaces with acoustic shadowing were seen. The abdominal aorta was visualized in 3 transverse images and a sagittal view. The aorta was measured in the anterior posterior plane, including the vessel wall, and diameter less than 3 cm was considered normal, coherent with Wanhainen’s recommendation [28]. The gallbladder was visualized in a longitudinal plane, and a sweep was recorded. The gallbladder wall was measured in the most slender site, with less than 4 mm considered normal. An intraluminal finding of 1 or more focuses with hyperechoic surface and acoustic shadowing was considered positive for cholecystolithiasis, in agreement with the definition by Popescu et al. [29]. The urinary bladder was measured, and its volume was calculated using the formula volume = length x width x height × 0.52, with volumes larger than 400 mL considered pathological [30].

Focused Lung Ultrasound (FLUS): Ultrasound was performed in the 8 anterolateral zones according to Volpicelli [31]. All 8 zones were assessed for lung sliding, lung pulse, B-lines in any view, visible lung parenchymal pathology, and visible pleural fluid. Interstitial syndrome was defined as 3 or more B-lines in more than 1 view. Parenchymal pathology was images without a clear pleural line and hypoechoic areas within the lung parenchyma. The findings were interpreted as being positive for pneumothorax if lung sliding was absent and a lung point was located.

All ultrasound examinations were performed by the principal investigator (JW) using a GE Vivid S6 (GE Healthcare, Chicago, Illinois, USA) ultrasound system with a M4S-RS 1.5–3.6 MHz phased array probe for cardiac views and a 4C-RS 1.8–6.0 MHz convex array (curved) for all other views. All views were recorded as cine-loops and exported in DICOM format to an external hard drive. The images underwent post-analysis in EchoPac (GE Healthcare, Chicago, Illinois, USA).

## Validity of the ultrasound examinations

To ensure sufficient competency, the primary investigator (JW) underwent three POCUS courses and performed more than 100 of each specific type of ultrasound scan included in the study before patient inclusion was initiated. This number was chosen because it is well beyond the 25–50 scans recommended by the American College of Emergency Physicians (ACEP) [32], and the learning curve for basic scans seems to reach equilibrium before 100 [19]. Lastly, JW went through specific certification overseen by 3 experts in different areas of POC ultrasonography: FLUS, FAUS (including FAST), and FoCUS.

After patient inclusion, scans from 50 patients were audited, with all 20 of their possible cine-loops, corresponding to approximately 1000 (12.3% of all) cine-loops. The patients were selected by random draw in 10 clusters of all included patients. Three specialists with more than 5 years of experience in performing and teaching POCUS and with a relevant PhD degree reviewed the cine-loops. All ultrasound cine-loops from patients selected for audit were anonymized and uploaded to a secure online service to allow review by the auditors. All loops were rated on a 5-point Likert scale, as recommended by the American College of Emergency Physicians (ACEP) for reporting ultrasound cine-loops [33]:

1. No recognizable structures, no objective data can be gathered.
2. Minimally recognizable structures but insufficient for diagnosis.
3. Minimal criteria met for diagnosis, recognizable structures but with some technical or other flaws.
4. Minimal criteria met for diagnosis, all structures imaged well, and diagnosis easily supported.
5. Minimal criteria met for diagnosis, all structures imaged with excellent image quality, and diagnosis completely supported.

After the audit, the results were dichotomized into 0 = insufficient for diagnosis (1 or 2) and 1 = sufficient for diagnosis (3, 4, or 5).

## Statistics

Descriptive data are presented as actual numbers and percentages. Normal distributions are presented as means with standard deviations. Normal distribution was assessed using Q-Q plots and histograms. In case of a nonnormal distribution, the data are presented as a median with interquartile range (IQR). The differences between multiple medians were calculated using the Kruskall-Wallis test. Agreement on image quality between the principal investigator and the auditors was calculated using Cohen’s kappa, and agreement on pathology is presented as percentages of agreement as suggested by, McHugh [34]. We used Landis and Koch’s guidelines for interpreting Cohen’s Kappa [35]. Data analysis was performed with Stata 13 (Statacorp, Texas).

## Results

**Fig. 2:**
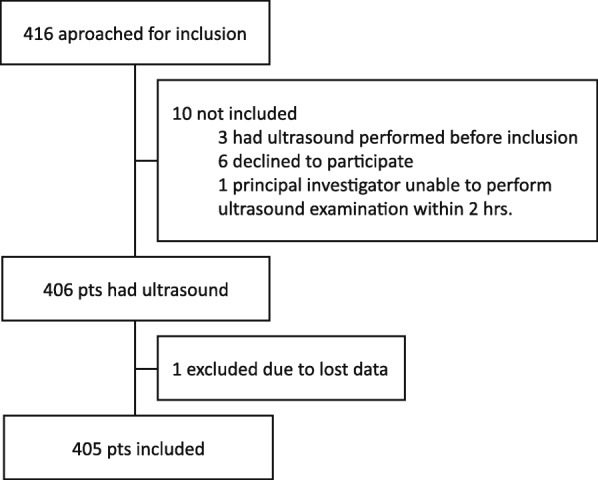
Trial Profile

From March 4, 2014, to February 23, 2016, 416 patients were screened for study participation, of whom 10 patients were excluded. Three had a FAST examination performed before inclusion, 6 declined to participate, and the primary investigator could not perform the examination within 2 h for 1 patient. A total of 406 patients had an ultrasound examination performed. One patient was subsequently excluded due to all cine-loops being lost between recording and exporting the files from the ultrasound machine. The trial profile is shown in Fig. 2. The final analysis included 405 patients. The baseline characteristics are shown in Table 1.

**Table 1:** Baseline Characteristics and Initial Vital Signs of Participants

Characteristic	Total (*n* = 405)
Age, median (IQR)	55.7 (38.5; 70.1)
Male n (%)	247 (61.0)
BMI, median (IQR)	25.4 (22.9; 29.0)
BP systolic, mean (SD)	142.5 (25.5)
BP diastolic, mean (SD)	85.0 (14.9)
Temperature, median (IQR)	37 (36.6; 37.3)
Respiration rate, median (IQR)	16 (16; 18)
Heart rate, mean (SD)	76,2 (17,6)
Heart rate, median (IQR)	75 (65; 87)
SpO2, median (IQR)	98 (96; 99)
Smoker n (%)	53 (13.1)
Alcohol abuse n (%)	16 (4.0)

*IQR* interquartile range, *BMI* body mass index, *BP* blood pressure, *SD* standard deviation, *SpO2 Peripheral oxygen saturation*

Stratification was applied to investigate the baseline characteristics among the orthopedic, medical, and abdominal surgical subcategories. This revealed a significant age difference, with orthopedic patients being youngest. The median ages (IQR) were: 51.8 (34.9; 68.1), 61.3 (44.5; 77.1), and 55.8 (37.8; 68.3), respectively, with a Kruskall-Wallis test producing *P* = .002. No other analysis revealed significant differences baseline characteristics among groups.

## Feasibility

Not all views were obtained for every patient; we obtained 94.5% of all possible ultrasound views. All patients had at least 1 cine-loop performed. The obtained images are detailed in Table 2. For extensive information on which images where obtained please see the Additional file 1: Table S9.

**Table 2:** Total of All Cine Loops Performed and Stored, n (%)

Sonographic window	Orthopedic (*n* = 191)	Medical (*n* = 120)	Abdominal Surgical (*n* = 94)	Total (*n* = 405)
Focused cardiac ultrasound views	887 (92.9)	570 (95.0)	447 (95.1)	1904 (94.0)
Focused abdominal ultrasound views	893 (93.5)	559 (93.2)	435 (92.6)	1887 (93.2)
FAST views	372 (97.4)	237 (98.8)	184 (97.9)	793 (97.9)
Focused lung ultrasound views	1439 (94.2)	921 (95.9)	712 (94.7)	3072 (94.8)
TOTAL performed/possible (%)	3591/3820 (94.01)	2287/2400 (95.30)	1778/1880 (94.57)	7656/8100 (94.51)

## Audit

The audit of 50 (12.3%) patients resulted in a Cohen’s kappa of 0.58 (moderate), with a corresponding percentage of agreement of 87.63% between the auditors’ vs. principal investigator’s scores on the 5-point Likert scale after it was dichotomized (as described in the Validity section). When analyzing for pathology versus no pathology, the percentage of agreement was 94.46%, with a corresponding Cohen’s kappa of 0.87 (almost perfect).

## Results from the ultrasound examinations

POCUS revealed 1 or more positive findings in 159 (39.3%; 95% CI, 34.5–44.0) out of 405 included patients. The subcategories are shown in Table 3. Table 4 also shows the distribution of positive findings among the different types of ultrasound examinations per categories of chief complaint. Cumulated positive findings in the abdominal, lung, cardiac, and FAST examinations were revealed 194 times in 159 (39.3%; 95% CI, 34.5–44.0) unique patients. Divided among the categories of chief complaint, we found 62 positive scans in 58 (14.3%; 95% CI, 10.9–17.7) unique patients with orthopedic complaints, 77 positive scans among 59 (14.6%; 95% CI, 11.1–18.0) unique patients with medical complaints, and 55 scans among 42 (10.4%; 95% CI, 7.4–13.3) unique patients with abdominal surgical complaints. (For in depth descriptions of findings see Additional file 1: Tables S1-S7). Kidney cysts were discovered in 24 (5.9%; 95% CI, 3.6–8.2) of all patients but were not considered positive findings since simple kidney cysts are a normal variation.

**Table 3:** Positive findings from POCUS on unselected patients in the emergency department listed by subcategory of examinations

Variables	Pts with positive findings
	n (%) [95% CI]
Positive findings per examinations in subcategories (*n* = 405)	Pts with positive findings
	n (%) [95% CI]
Focused Abdominal Ultrasound (FAUS)	
Cholecystolithiasis	42 (10.4) [7.4–13.3]
Large urinary bladder (> 400 mL)	40 (9.9) [7.0–12.8]
Abdominal Aortic Aneurism	10 (2.5) [0.1–4.0]
Free Fluid (FAST)	15 (3.7) [1.8–5.6]
Hydronephrosis	8 (2.0) [0.1–3.3]
Focused Lung Ultrasound (FLUS)	
Pulmonary effusion	27 (6.7) [1.0–9.1]
Parenchymal pathology	19 (4.7) [2.6–6.8]
Localized b-lines	12 (3.0) [1.3–4.6]
Interstitial syndrome	11 (2.7) [1.1–4.3]
Pneumothorax	3 (0.7) [−0.0–1.6]
Focused Cardiac Ultrasound (FoCUS)	
Reduced LV contractility	19 (4.7) [2.6–6.8]
Pericardial effusion	9 (2.2) [0.8–3.7]
Right ventricular dilation	3 (0.7) [−0.0–1.6]

*Pts* patients, *LV* Left ventricle, *FAST* Focused assessment with sonography in trauma

**Table 4:** Positive findings from POCUS on unselected patients in the emergency department listed by subcategory of examinations per category of chief complaint

All findings, patients n (%) [95% CI]				
Examination type	Orthopedic complaint	Medical complaint	Surgical complaint	Total
	(*n* = 191)	(*n* = 120)	(*n* = 94)	(*n* = 405)
Focused Cardiac Ultrasound (FoCUS)	10 (5.24) [2.0–8.4]	9 (7.50) [2.8–12.2]	9 (9.57) [3.5–15.6]	28 (6.91) [4.4–9.4]
Focused Lung Ultrasound (FLUS)	17 (8.90) [4.8–13.0]	31 (25.83) [17.9–33.8]	15 (15.96) [8.4–23.5]	63 (15.56) [12.0–19.1]
Focused Abdominal Ultrasound (FAUS)	32 (16.8) [11.4–22.0]	30 (25.0) [17.2–32.8]	26 (27.7) [18.4–36.9]	88 (21.7) [17.7–25.8]
Focused Assessment With Sonography in Trauma (FAST)	3 (1.57) [− 0.2–3.4]	7 (5.83) [1.6–10.1]	5 (5.32) [0.7–9.9]	15 (3.70) [1.9–5.6]
Unique patients	58 (30.4) [23.8–36.9]	59 (49.2) [40.1–58.2]	42 (44.7) [34.4–54.9]	159 (39.3) [34.5–44.0]

## Discussion

In this exploratory prospective study of 405 unselected patients in the ED, positive ultrasound findings could be identified in more than every third patient when whole-body POCUS was performed. The magnitude of this proportion is remarkable.

This study contributes to the general knowledge of the prevalence of ultrasound findings, which will be important for any clinician performing ultrasound. The revealed pathologies are all based on basic ultrasound examinations. This knowledge is paramount if pre- and posttest probability should be evaluated before the physician’s final synthesis of any patient presentation [8].

A recent cross-sectional study by Bobbia et al. found that in 1 day in 50 EDs in France, 192 of 4671 total patients (4%) had an ultrasound performed [36]. The present study revealing positive findings in 39.3% of all patients, which leaves the impression that the total potential of POCUS is not fully exploited in everyday praxis. POCUS is still merely applied to critically ill patients of patients for whom the physician expects benefit. However, the remarkably high number of pathological findings in this study should raise curiosity about expanding its current use.

Focused abdominal ultrasound revealed most of the findings in this study. The 2 most present positive findings were cholecystolithiasis and large urinary bladder. Both are notorious for being present without clinical impact or importance but can also potentially cause a fatal disease. Future research will be needed to investigate the clinical importance of these findings, as the present study did not analyze the clinical relevance of the pathological findings.

When investigating categories of chief complaints, the patients with orthopedic complaints were younger, ultrasound revealed fewer positive findings, and a large percentage of the patients with any positive finding had only 1 finding. This can be interpreted as indicating that these patients would have the least benefit from POCUS. However, more research is needed to elucidate this.

Using POCUS as a screening tool may lead to the early detection of occult pathology and early treatment. In the present study, we did not investigate the level of importance of the pathology or its relation to the presentation symptoms; hence, we can only conjecture that POCUS made a significant difference to these patients. It is well known that not all positive ultrasound findings require treatment or have any relation to the clinical presentation in the ED. Thus, there is a risk of overdiagnostication, resulting in misuse of CT or X-ray, with potential harmful effects such as radiation, discomfort, time delay, or even unnecessary treatment with possible adverse effects. Overdiagnostication can also lead to superfluous concern, as seen in other screening programs [37]. If the POCUS adds unnecessary information to the patient’s presentation that is ambiguous to the physician, the physician must be able to put the scans into clinical context [38]. The present study does not support using whole-body POCUS on all patients, but promotes the idea that more patients might benefit from ultrasound than are currently being examined.

This study generates hypotheses, and we encourage future research to focus on the actual relevance of any positive finding revealed by POCUS. Future research questions arise:

Do the findings induce changes in the patient treatment or workup? Which clinical presentations have the largest benefit from ultrasound, in terms of both severity (such as triage level) and chief complaint? Answering these questions will assist the physician with applying POCUS on the right patients at the right time. The study agrees with the notion that POCUS should be focused and based on clinical presentation. The knowledge produced will assist the physician performing focused ultrasound to interpret if any finding is rare or common.

## Limitations

The study is associated with some limitations. The principal investigator (JW) performed all of the POCUS examinations. First, it can be questioned if the principal investigator had sufficient expertise. Yet, the audit of 1000 cine-loops did show a high level of agreement upon pathology of 94.46%, with a corresponding Cohen’s kappa of 0.87. The latter was interpreted as strong [29]. Second, this might reduce the external validity, as it can be speculated that the level of competence among physicians in the ED is not as high as the competence of the expert performing the scans in this study. This is a concern, as ultrasound is regarded as being highly operator dependent [39]. However, having only performed 100 of each type of ultrasound examination, the primary investigator was not an expert when the study was initiated. This is a feasible number of examinations for any emergency physician to obtain; therefore, the study findings are somewhat transferable to the level of expertise of any emergency physician using ultrasound in his or her everyday praxis.

In some geographical areas the patient characteristics such as body mass index might differ from this study making ultrasound either more or less likely to identify abnormalities. This should be taken into consideration when interpreting the findings in a local setting.

## Conclusion

This study presents the prevalence of positive findings revealed by performing basic whole-body ultrasound on unselected patients. Positive findings were revealed in 39.3% of all included patients. The potential clinical impact of these findings is unclear and should be explored in future research.

## Additional file

Additional file 1: The supplementary file includes subgroup results from ultrasound examinations, list of keywords for three main categories of complaints or clinical presentations, and an overview of the total scans performed. (DOCX 120 kb)

## Abbreviations

BMUS: British Medical Ultrasound Society
CT: Computer tomography
ED: Emergency Department
FAST: Focused assessment with sonography in trauma
FOCUS: Point-of-care ultrasound
GP: General practitioner

## References

[1] Bernstein SL (2009). The effect of emergency department crowding on clinically oriented outcomes. Acad Emerg Med.

[2] Campbell SJ, Bechara R, Islam S (2018). Point-of-care ultrasound in the intensive care unit. Clin Chest Med.

[3] Moore CL, Copel JA (2011). Point-of-care ultrasonography. N Engl J Med.

[4] Blaivas M (2001). Triage in the trauma bay with the focused abdominal sonography for trauma (FAST) examination. J Emerg Med.

[5] Mandavia DP, Hoffner RJ, Mahaney K, Henderson SO (2001). Bedside echocardiography by emergency physicians. Ann Emerg Med.

[6] Jones AE, Tayal VS, Sullivan DM, Kline JA (2004). Randomized, controlled trial of immediate versus delayed goal-directed ultrasound to identify the cause of nontraumatic hypotension in emergency department patients. Crit Care Med.

[7] Hwang JQ, Kimberly HH, Liteplo AS, Sajed D (2011). An evidence-based approach to emergency ultrasound. Emerg Med Pract.

[8] Doust J (2009). Diagnosis in general practice. Using probabilistic reasoning. BMJ.

[9] Ma OJ (1995). Prospective analysis of a rapid trauma ultrasound examination performed by emergency physicians. J Trauma.

[10] Laursen CB (2014). Point-of-care ultrasonography in patients admitted with respiratory symptoms: a single-blind, randomised controlled trial. Lancet Respir Med.

[11] Lichtenstein DA (2005). Ultrasound diagnosis of occult pneumothorax. Crit Care Med.

[12] Xirouchaki N, Kondili E, Prinianakis G, Malliotakis P, Georgopoulos D (2014). Impact of lung ultrasound on clinical decision making in critically ill patients. Intensive Care Med.

[13] Lichtenstein DA, Meziere GA (2008). Relevance of lung ultrasound in the diagnosis of acute respiratory failure: the BLUE protocol. Chest.

[14] S-Hariri B (2015). The impact of using RUSH protocol for diagnosing the type of unknown shock in the emergency department. Emerg Radiol.

[15] Olejaz M (2012). Denmark health system review. Health Syst Transit.

[16] Haahr M. True Random Number Service. www.random.org. Published 1998. Accessed Jan 2014.

[17] Atkinson P (2015). International Federation for Emergency Medicine point of care ultrasound curriculum. CJEM.

[18] Laursen CB (2014). A framework for implementation, education, research and clinical use of ultrasound in emergency departments by the Danish society for emergency medicine. Scand J Trauma Resusc Emerg Med.

[19] Blehar DJ, Barton B, Gaspari RJ (2015). Learning curves in emergency ultrasound education. Acad Emerg Med.

[20] Seif D, Perera P, Mailhot T, Riley D, Mandavia D. Bedside ultrasound in resuscitation and the rapid ultrasound in shock protocol. Crit Care Res Pract. 2012;503254. 10.1155/2012/503254 PMC3485910 23133747

[21] Breitkreutz R (2010). Focused echocardiographic evaluation in life support and peri-resuscitation of emergency patients: a prospective trial. Resuscitation.

[22] Jensen MB, Sloth E, Larsen KM, Schmidt MB (2004). Transthoracic echocardiography for cardiopulmonary monitoring in intensive care. Eur J Anaesthesiol.

[23] Labovitz AJ (2010). Focused cardiac ultrasound in the emergent setting: a consensus statement of the American Society of Echocardiography and American College of Emergency Physicians. J Am Soc Echocardiogr.

[24] Fremont B (2008). Prognostic value of echocardiographic right/left ventricular end-diastolic diameter ratio in patients with acute pulmonary embolism: results from a monocenter registry of 1,416 patients. Chest.

[25] Henwood PC (2017). Point-of-care ultrasound use, accuracy, and impact on clinical decision making in Rwanda hospitals. J Ultrasound Med.

[26] Scalea TM (1999). Focused assessment with sonography for trauma (FAST): results from an international consensus conference. J Trauma.

[27] Dalziel PJ, Noble VE (2013). Bedside ultrasound and the assessment of renal colic: a review. Emerg Med J.

[28] Wanhainen A (2008). How to define an abdominal aortic aneurysm--influence on epidemiology and clinical practice. Scand J Surg.

[29] Popescu A, Sporea I (2010). Ultrasound examination of normal gall bladder and biliary system. Med Ultrason.

[30] Dicuio M (2005). Measurements of urinary bladder volume: comparison of five ultrasound calculation methods in volunteers. Arch Ital Urol Androl.

[31] Volpicelli G (2006). Bedside lung ultrasound in the assessment of alveolar-interstitial syndrome. Am J Emerg Med.

[32] American College of Emergency Physicians (2009). Emergency ultrasound guidelines. Ann Emerg Med.

[33] American College of Emergency Physicians (2011). Emergency ultrasound standard reporting guidelines.

[34] McHugh ML (2012). Interrater reliability: the kappa statistic. Biochem Med (Zagreb).

[35] Landis JR, Koch GG (1977). An application of hierarchical kappa-type statistics in the assessment of majority agreement among multiple observers. Biometrics.

[36] Bobbia X (2017). The clinical impact and prevalence of emergency point-of-care ultrasound: a prospective multicenter study. Anaesth Crit Care Pain Med.

[37] Gotzsche PC, Jorgensen KJ. Screening for breast cancer with mammography. Cochrane Database Syst Rev. 2013:CD001877. 10.1002/14651858.CD001877.pub5 PMC6464778 23737396

[38] Bahner DP, Hughes D, Royall NA (2012). I-AIM: a novel model for teaching and performing focused sonography. J Ultrasound Med.

[39] Pinto A (2013). Sources of error in emergency ultrasonography. Crit Ultrasound J.

[40] Society and College of Radiographers and British Medical Ultrasound Society. Society, Guidelines for Professional Ultrasound Practice. www.bmus.org. Published 2015. Accessed August 2015.

