# Supplementary material for: Point-of-care ultrasound findings in unselected patients in an emergency department —results from a prospective observational trial
Source: BMC Emerg Med. 2018 Dec 27;18:60. doi: 10.1186/s12873-018-0211-4 (PMC6307264; doi:10.1186/s12873-018-0211-4)
Supplement: Supplementary file 1 — The supplementary file includes subgroup results from ultrasound examinations, list of keywords for three main categories of complaints or clinical presentations, and an overview of the total scans performed. (DOCX 120 kb) [file 12873_2018_211_MOESM1_ESM.docx]

**Subgroup results from ultrasound examinations**

**Focused Cardiac Ultrasound findings**

Unique patients with pathology on focused cardiac ultrasound (reduced left ventricular function, pericardial effusion and/or dilated right ventricle): n(%): 28 (6,91))

Table 1: Left ventricular contractility assessed by eyeballing - n(%).

|  | **Hyperdynamic** | **Normal** | **Mildly reduced** | **Moderately reduced** | **Severely reduced** | **Unable to assess** |
| --- | --- | --- | --- | --- | --- | --- |
| **Orthopedic (n=191)** | 3 (1,57) | 168 (87,96) | 3 (1,57) | 5 (2,62) | 0 (0,00) | 12 (6,28) |
| **Medical (n=120)** | 12 (10,00) | 97 (80,83) | 0 (0,00) | 3 (2,50) | 2 (1,67) | 6 (5,00) |
| **Surgical (n=94)** | 3 (3,19) | 83 (88,30) | 2 (2,13) | 3 (3,19) | 1 (1,06) | 2 (2,13) |
| Total | 18 (4,44) | 348 (85.93) | 5 (1,23) | 11 (2,72) | 3 (0,74) | 20 (4,94) |

Table 2: Pericardial effusion and right ventricular dilation - n(%).

**Pericardial effusion Right ventricular dilation**

|  | **Yes** | **No** | **Unable to assess** | **Yes** | **No** | **Unable to assess** |
| --- | --- | --- | --- | --- | --- | --- |
| **Orthopedic (n=191)** | 2 (1,05) | 182 (95,29) | 7 (3,66) | 2 (1,05) | 181 (94,76) | 8 (4,19) |
| **Medical (n=120)** | 4 (3,33) | 113 (94,17) | 3 (2,50) | 1 (0,83) | 117 (97,50) | 2 (1,67) |
| **Surgical (n=94)** | 3 (3,19) | 91 (96,81) | 0 (0,00) | 0 (0,00) | 91 (96,81) | 3 (3,19) |
| **Total (n=405)** | 9 (2,22) | 386 (95,31) | 10 (2,47) | 3 (0,74) | 389 (96,05) | 13 (3,21) |

**Focused Lung Ultrasound findings**

Unique patients with pathology on focused lung ultrasound (Patients with either pulmonary effusion, interstitial syndrome, parenchymal pathology, localized b-lines and/or pneumothorax) n(%): 63 (15,56)

Table 3: **Pulmonary effusion Interstitial syndrome**

|  | **Yes** | **No** | **Unable to assess** | **Yes** | **No** | **Unable to assess** |
| --- | --- | --- | --- | --- | --- | --- |
| **Orthopedic (n=191)** | 5 (2,62) | 181 (94,76) | 5 (2,62) | 2 (1,05) | 185 (96,86) | 4 (2,09) |
| **Medical (n=120)** | 15 (12,50) | 102 (85,00) | 3 (2,50) | 9 (7,50) | 108 (90,00) | 3 (2,50) |
| **Surgical (n=94)** | 7 (7,45) | 86 (91,49) | 1 (1,06) | 0 (0,00) | 93 (98,94) | 1 (1,06) |
| **Total (n=405)** | 27 (6,67) | 369 (91,11) | 9 (2,22) | 11 (2,72) | 386 (95,31) | 8 (1,98) |

Table 4: **Consolidated lung Localized b-lines**

|  | **Yes** | **No** | **Unable to assess** | **Yes** | **No** | **Unable to assess** |
| --- | --- | --- | --- | --- | --- | --- |
| **Orthopedic (n=191)** | 2 (1,05) | 181 (94,76) | 8 (4,19) | 0 (0,00) | 183 (95,81) | 8 (4,19) |
| **Medical (n=120)** | 12 (10,00) | 107 (89,17) | 1 (0,83) | 8 (6,67) | 111 (92,50) | 1 (0,83) |
| **Surgical (n=94)** | 5 (5,32) | 88 (93,62) | 1 (1,06) | 4 (4,26) | 89 (94,68) | 1 (1,06) |
| **Total (n=405)** | 19 (4,69) | 376 (92,84) | 10 (2,47) | 12 (2,96) | 383 (94,57) | 10 (2,47) |

Table 5: **Pneumothorax**

|  | **Yes** | **No** | **Unable to assess** |
| --- | --- | --- | --- |
| **Orthopedic (n=191)** | 1 (0,52) | 180 (94,24) | 10 (5,24) |
| **Medical (n=120)** | 2 (1,67) | 113 (94,17) | 5(4,17) |
| **Surgical (n=94)** | 0 (0,00) | 91 (96,81) | 3 (3,19) |
| **Total (n=405)** | 3 (0,74) | 384 (94,81) | 18 (4,44) |

**Table 6: FAST (Focused Assessment with Sonography in Trauma) findings**

Patients with intraperitoneal fluid - n(%).

**Free Fluid**

|  | **Yes** | **No** | **Unable to assess** |
| --- | --- | --- | --- |
| **Orthopedic (n=191)** | 3 (1,57) | 179 (93,72) | 9 (4,71) |
| **Medical (n=120)** | 7 (5,83) | 112 (93,33) | 1 (0,83) |
| **Surgical (n=94)** | 5 (5,32) | 86 (91,49) | 3 (3,19) |
| **Total (n=405)** | 15 (3,70) | 377 (93,09) | 13 (3,21) |

**Table 7: Focused Abdominal Ultrasound Findings**

Sorted into specific findings per subgroup - n(%).

|  | **Hydronephrosis** | **Kidney Cysts** | **Abdominal Aortic Aneurism** | **Cholecystolithiasis** | **Large bladder (>400ml)** |
| --- | --- | --- | --- | --- | --- |
| **Orthopedic (n=191)** | 2 (1,05) | 6 (3,14) | 4 (2,09) | 10 (5,24) | 21 (10,99) |
| **Medical (n=120)** | 2 (1,67) | 12 (10,00) | 3 (2,50) | 17 (14,17) | 12 (10,00) |
| **Surgical (n=94)** | 4 (4,26) | 6 (6,38) | 3 (3,19) | 15 (15,96) | 7 (7,45) |
| **Unable to assess (n=405)** | 21 (5,12) | 21 (5,12) | 28 (6,91) | 49 (12,10) | 40 (9,88) |
| **Total (n=405)** | 8 (1,98) | 24 (5,93) | 10 (2,47) | 42 (10,37) | 40 (9,88) |

**Table 8: List of keywords for three categories of complaints or clinical presentations**

These where the words placing the patients in either category:

**Orthopedic complaints**

| Left shoulder | |
| --- | --- |
| Left arm |  |
| Left elbow |  |
| Left antebrachium | |
| Left wrist | |
| Left hand |  |
| Left 1st finger | |
| Left 2^nd^ finger | |
| Left 3rd finger | |
| Left 5th finger | |
| Left hip |  |
| Left leg |  |
| Left knee |  |
| Left crus |  |
| Left ankle | |
| Left foot |  |
| Left 1st Toe |  |
| Left 5th toe |  |
|  |  |
|  |  |
|  |  |
| Right clavicle | |
| Right Shoulder | |
| Right arm |  |
| Right elbow |  |
| Right antebrachium | |
| Right wrist | |
| Right hand |  |
| Right 1st finger | |
| Right 2nd finger | |
| Right 3rd finger | |
| Right 4th finger | |
| Right 5th finger | |
| Right hip | |
| Right leg |  |
| Right thigh |  |
| Right knee |  |
| Right crus | |
| Rightright ancle | |
| Right foot |  |
| Right 1^st^ toe |  |
| Right 4^th^ toe |  |
| Right 5^th^ toe |  |
|  |  |
|  |  |
|  |  |
| Observation for concussion | |
| Back pain | |
| Traffic accident |  |
| Bike accident |  |
| Fall |  |
| Fall from horse | |
| Trauma |  |
| Assult |  |
| Neckpain | |
| Trampoline injury |  |
| Neck pain after traffic injury | |
| Lumbar spine pain after fall | |
| Fall from 1.5 meter | |
| Syncope after traffic accident | |
| Tendon lesion | |
| Burns | |
| Cuts |  |
| Trauma from squeeze | |
| Pneumothorax | |
| Scalding |  |
| Wound |  |
| Splinter in the eye | |
| Wound in the forehead | |
| Pearl i the nose | |
| Face |  |
| Accident to mounth | |
| Nose fracture | |
| Laceration in lip | |
| Walker |  |
| Mitella |  |
| Iriitation from splint |  |
| Clavicle |  |
| Shoulder pain | |
| Arm |  |
| Elbow |  |
| Colles fracture | |
| Wrist |  |
| Hand |  |
| Finger |  |
| 1st finger |  |
| 2^nd^ and 3rd finger | |
| 4th finger |  |
| 5th finger |  |
| 5th metacarpal | |
| Hip pain | |
| Leg |  |
| Knee | |
| Patella lux |  |
| Crus |  |
| Achilles tendon | |
| Distortion trauma | |
| Ancle |  |
| Inschemia right foot | |
| Foreign object in foot | |
| 1st toe |  |
| 4th toe |  |
| 2nd metatarsal | |

**Medical complaints:**

| Psychiatric examination |
| --- |
| Suicide attempt |
| Fever |
| Comatose |
| Migraine |
| DVT |
| Pneumonia |
| Headache |
| In need of transfusion |
| Hypotension |
| impaired consciousness |
| Leg swelling |
| Hemiparesis |
| Icterus |
|  |
| AFIB |
| Aphasia |
| Erysipelas |
| Hypokalemia |
| Hepatitis |
| Pleural effusion |
| Aortic aneurism |
| Ascites |
| Observation for high intracranial pressure |
| Mistake full fix |
| Cut on sharp object |
| COPD exacerbation |
| Hypoglycemia |
| Confusion |
| Electrocution |
| Tired |
| Vertigo |
| Low hgb |
| INR high |
| Asthma |
| Infection |
| Convulsions |
| Alcohol intoxication |
| Sclerosis |
| Incompensatio |
| Dyspnea |
| Chest pain |
| Lipotymi |
| Dehydration |
| Allergic reaction |
| Generally unwell Unwell |
| Unspecific |
| Respirational pain |

**Surgical complaints:**

| OB/GYN complaint |
| --- |
| Complaint associated to pregnancy |
| Vaginal bleed |
| Torc ovarian cyste |
| Ekstra uterine pregnancy |
| Endometrioses |
| Spontaneous abortion |
| Fever after abortion |
|  |
|  |
|  |
| Abdominal pain |
| Infection after operation |
| irreducible hernia |
| melena |
| Acute abdomen |
| Right flank pain |
| Pancreatitis |
| White bowel |
| diarrhea |
| Femoral hernia |
| Appendicitis |
| ulcers |
| Cholelithiasis |
| Rectal bleed |
| Wound needs tend |
| Obstipation |
| Sub ileus |
| Postoperative complication  Swell in groin |
| Coffee ground like emesis |
| Hematemesis |
| Swelling of the scrotum |
| Emesis |
| Perianal abscess |
| Pain right fossa |
| Bleeding |
| Hernia |
| Hemoptysis |
| Unable to swallow |
| Pain along right rib edge |
| Pyelonephritis |
| Hemorrhoid |
| Obstipation |
| Arterial bleed |
| Swallowed needle |
| Swallowed battery |
| Ischemia lower extremity |
| Arterial embolus lower extremity |
| Abscess us |
|  |

**Table 9: Total of all scans performed and stored n(%)**

| **Sonographic window** | **Orthopedic (n=191)** | **Medical (n=120)** | **Surgical (n=94)** | **Total (405)** |
| --- | --- | --- | --- | --- |
| **Cardiac views** |  |  |  |  |
| Subcostal 4 chamber | 185 (96,86) | 117 (97,50) | 92 (97,8) | 394 (97,28) |
| Parasternal long axis | 180 (94,24) | 117 (97,50) | 91 (96,81) | 388 (95,80) |
| Parasternal short axis | 175 (91,62) | 112 (93,33) | 88 (93,62) | 375 (92,59) |
| Apical 4 chamber | 177 (92,67) | 115 (95,83) | 90 (95,74) | 382 (94,32) |
| Inferior vena cava | 170 (89,01) | 109 (90,83) | 86 (91,49) | 365 (90,12) |
| **Abdominal views** |  |  |  |  |
| Galbladder | 160 (83,77) | 108 (90,00) | 88 (93,62) | 356 (87,90) |
| Right kidney | 186 (97,38) | 116 (96,67) | 88 (93,62) | 390 (96,30) |
| Left kidney | 185 (96,86) | 116 (96,67) | 91 (96,81) | 392 (96,79) |
| Aorta | 186 (97,38) | 113 (94,17) | 85 (90,43) | 384 (94,81) |
| Urinary bladder (2 views) | 176 (92,15) | 106 (88,33) | 83 (88,30) | 365 (90,12) |
| **FAST** |  |  |  |  |
| Right upper quadrant | 186 (97,38) | 119 (99,17) | 93 (98,94) | 398 (98,27) |
| Left upper quadrant | 186 (97,38) | 118 (98,33) | 91 (96,81) | 395 (97,53) |
| **Lungs** |  |  |  |  |
| Volpicelli 1 | 183 (95,81) | 115 (95,83) | 91 (96,81) | 389 (96,05) |
| Volpicelli 2 | 182 (95,29) | 115 (95,83) | 89 (94,68) | 386 (95,31) |
| Volpicelli 3 | 168 (87,96) | 112 (93,33) | 86 (91,49) | 366 (90,37) |
| Volpicelli 4 | 188 (98,43) | 118 (98,33) | 91 (96,81) | 397 (98,02) |
| Volpicelli 5 | 183 (95,81) | 115 (95,83) | 91 (96,81) | 389 (96,05) |
| Volpicelli 6 | 179 (93,72) | 114 (95,00) | 87 (92,55) | 380 (93,83) |
| Volpicelli 7 | 169 (88,48) | 113 (94,17) | 87 (92,55) | 369 (91,11) |
| Volpicelli 8 | 187 (97,91) | 119 (99,17) | 90 (95,74) | 396 (97,78) |
| TOTAL performed/possible (%) | 3591/3820 (94,01) | 2287/2400 (95,30) | 1778/1880 (94,57) | 7656/8100(94,51) |
